# Effects of maturation on knee biomechanics during cutting and landing in young female soccer players

**DOI:** 10.1371/journal.pone.0233701

**Published:** 2020-05-26

**Authors:** Audrey E. Westbrook, Jeffrey B. Taylor, Anh-Dung Nguyen, Mark V. Paterno, Kevin R. Ford

**Affiliations:** 1 Department of Physical Therapy, High Point University, High Point, North Carolina, United States of America; 2 Department of Physical Therapy, The University of Tennessee Health Science Center, Memphis, Tennessee, United States of America; 3 Division of Athletic Training, West Virginia University, Morgantown, West Virginia, United States of America; 4 Division of Occupational Therapy and Physical Therapy, Division of Sports Medicine Cincinnati Children’s Hospital Medical Center, Department of Pediatrics, University of Cincinnati College of Medicine, Cincinnati, Ohio, United States of America; Mayo Clinic Rochester, UNITED STATES

## Abstract

Young female soccer players are at high risk of anterior cruciate ligament injury due to the fast-paced nature of the sport and surplus of unplanned movements during play. Neuromuscular training programs that aim to reduce this injury by targeting the associated biomechanical movements are a potential solution. While previous studies have examined the lack of dynamic knee control during landing, there are few that outline the role that maturation can play during unanticipated cutting. Therefore, the purpose of this study was to determine if young female soccer players across multiple phases of maturation exhibited the before seen differences in knee control during a drop landing as well as an unanticipated cutting task. 139 female soccer players volunteered to participate in this study and were classified in three maturational groups based on percent adult stature: prepubertal (PRE), pubertal (PUB), and post-pubertal (POST). Each group performed a drop vertical jump (DVJ) and an unanticipated cutting task (CUT). Standard 3D motion capture techniques were used to determine peak knee flexion/abduction angles and moments during each task. Within tasks, POST exhibited significantly greater peak abduction angles and moments compared to PUB/PRE. While each maturational group exhibited greater peak knee abduction angles during the DVJ compared to the CUT, peak knee abduction moments during the CUT were greater compared to the DVJ. Participants within each maturational group exhibited greater knee flexion during the DVJ compared to the CUT, however there were no differences identified between groups. During both tasks, POST/PUB exhibited greater peak knee flexion moments compared to PRE, as well as POST compared to PUB. Overall, each group exhibited significantly greater peak knee flexion moments during the CUT compared to the DVJ. These observed differences indicate the need for neuromuscular training programs that target both jumping and cutting techniques to reduce ACL injuries.

## Introduction

Injuries in young female athletes are an increasing heath problem within the United States [1]. In sports like soccer, accelerating, decelerating, and performing multidirectional movements are common. Unfortunately, due in part to these fast-paced sport-related tasks, injuries are prevalent and tend to occur in the lower extremities [2]. Of specific interest are non-contact injuries which often involve unplanned or reactive movements (i.e. jumping, landing, cutting) without a direct blow (i.e. collision, tackling) [3]. The incidence of anterior cruciate ligament (ACL) injuries in adolescent athletes has continued to climb over the last 20 years, with females showing the greatest increases [4]. A meta-analysis showed that as female athletes reach their mid-teens, sports related ACL injuries are continuing to increase, indicating a potential optimal window for injury prevention interventions [5]. Specifically, neuromuscular training programs are a potential solution that can address these types of injuries, especially those that target the high-risk biomechanical movements associated with injury [6, 7].

Studies have identified that knee motion and joint moments during dynamic sport related activities may be the most important movement strategy to target as a risk factor for ACL injury [8, 9]. During the adolescent growth spurt, increases in an athlete’s height and mass with longer lever arms of the femur and tibia can potentially increase the torque (moment) at that joint. While measurements like peak knee abduction moment increase for both males and females during adolescent growth, only males exhibit a recovery of dynamic knee control after reaching near adult stature [10]. Females, in contrast, continue to show a decrease in dynamic knee control combined with significant increases in peak knee abduction moments as they reach late adolescent stages, potentially contributing to increased risk of ACL injury [11]. Focusing on these specific mechanisms of injury during maturational development of female athletes can help identify those most at risk.

Both double-leg landing and single-leg deceleration/pivoting tasks are often incorporated into assessments of movement quality. While evidence supports increases in knee abduction during double leg drop vertical jumps in adolescent female athletes immediately following the rapid adolescent growth spurt compared to males [10, 11], investigations are lacking that report the effects of maturation on knee abduction and joint moment during unanticipated cutting, which may be a common mechanism of injury [7]. The purpose of this study was to determine if adolescent female soccer players across multiple phases of maturation exhibited biomechanical differences in knee control during two dynamic, sport-related tasks. It was hypothesized that post-pubertal athletes would exhibit greater knee abduction angle and joint moment during each task compared to prepubertal and pubertal athletes.

## Materials and methods

### Participants

One hundred and thirty-eight young female soccer players volunteered to participate in this study (N = 138; Age: 13.5 ± 2.1 years; Height: 157.0 ± 10.4 cm; Mass: 50.6 ± 11.2 kg; Race: American Indian: n = 2, Asian: n = 3, Black: n = 1, White: n = 126, Multiple Races: n = 5, Unknown: n = 1; Ethnicity: Hispanic: n = 15, Not Hispanic: n = 119, Unknown: n = 4). Participants were included in this study if they were a female soccer player between the ages of 9 and 19 and able to participate in sport with no reported injury at the time of enrollment. Previous history of injury was not considered for inclusion criteria but was recorded following enrollment. All participants were informed of study benefits and risks. This study was approved by the High Point University Institutional Review Board. Participants that were 18 or older provided a signed written informed consent to participate in this study. Participants that were under 18 provided a signed written child assent and a signed written parental permission from a parent or guardian.

### Procedures

Each participant completed an electronic REDCap (Research Electronic Data Capture) [12] survey to determine demographic information and parental heights for prediction of percent adult stature using the Khamis-Roche method [11, 13]. Prepubertal (PRE) participants were classified as less than 87% adult stature (PRE: n = 17, 84.0 ± 1.8% adult stature, 10.3 ± 0.6 years, 137.8 ± 6.8 cm, 34.2 ± 4.5 kg), pubertal (PUB) from 87 to 94% adult stature (PUB: n = 32, 91.2 ± 2.1% adult stature 11.9 ± 0.8 years, 151.1 ± 5.7 cm, 43.3 ± 6.0 kg), and post-pubertal (POST) as greater than 94% adult stature (POST: n = 90, 97.9 ± 1.7% adult stature 14.6 ± 1.6 years, 162.6 ± 5.6 cm, 56.2 ± 8.8 kg) [10, 11]. Limb dominance was determined with the question: “If you were to kick a ball as far as you could, what leg would you choose to kick with?"

Lower extremity biomechanics were collected during a bilateral drop vertical jump (DVJ) and a single-leg unanticipated cutting task (CUT) [14]. Participants were instrumented with 43 retroreflective markers placed on the sternum, sacrum, left posterior superior iliac spine, C7, 3 points on the upper back (via a thin backpack), and bilaterally on the shoulder, upper arm, elbow, wrist, anterior superior iliac spine, greater trochanter, midthigh, medial and lateral knee joint line, tibial tubercle, midshank, distal shank, medial and lateral malleolus, and to the foot at the heel, dorsal surface of the lateral midfoot, lateral rear foot and 2nd met via double-sided tape [15]. Participants performed these tasks while wearing laboratory provided standardized cleated footwear (adidas x15.2; Beaverton, Oregon, USA) over a synthetic turf embedded with two force platforms (AMTI). Kinetic data were sampled at 1200 Hz from the force platforms while three-dimensional data were sampled at 200 Hz using a 15-camera motion analysis system (Cortex v7; Motion Analysis Corp, Santa Rosa, California, USA). A static trial was collected to determine neutral alignment and anatomical body segment definitions by which biomechanical measures were referenced. Biomechanical data were collected following a self-selected warm up and 1–2 practice trials for each task.

#### DVJ

**Fig 1:**
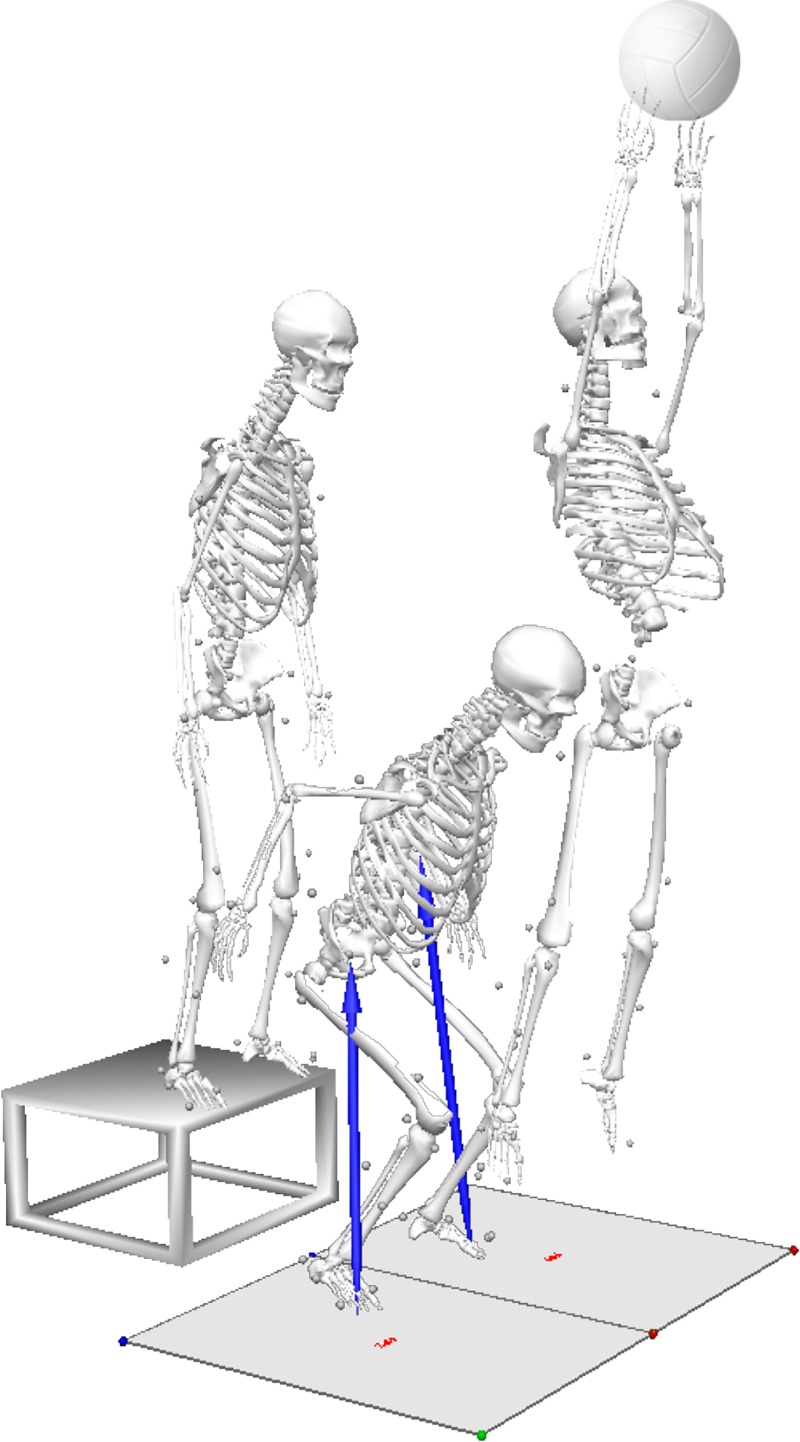
Drop vertical jump. Example participant performing the drop vertical jumping task.

A target was set for each participant using a maximal double-leg countermovement jump. After being instructed to jump as high as possible, the target (a ball) was suspended at a height where participants could barely touch with their fingertips. Once set, participants completed three trials of a DVJ that began with participants at arms by their sides and feet positioned 35-cm apart on top of a 31-cm high box. They were instructed to drop down from the box, leaving with both feet at the same time, land and then immediately perform a maximal vertical jump while reaching for the target with both hands (Fig 1) [9]. There were no limitations placed on the second landing. A trial was repeated if the participants did not leave the box with both feet at the same time, if they did not reach for the target with both hands, or if they did not land cleanly on the force platforms (one foot in each).

#### CUT

**Fig 2:**
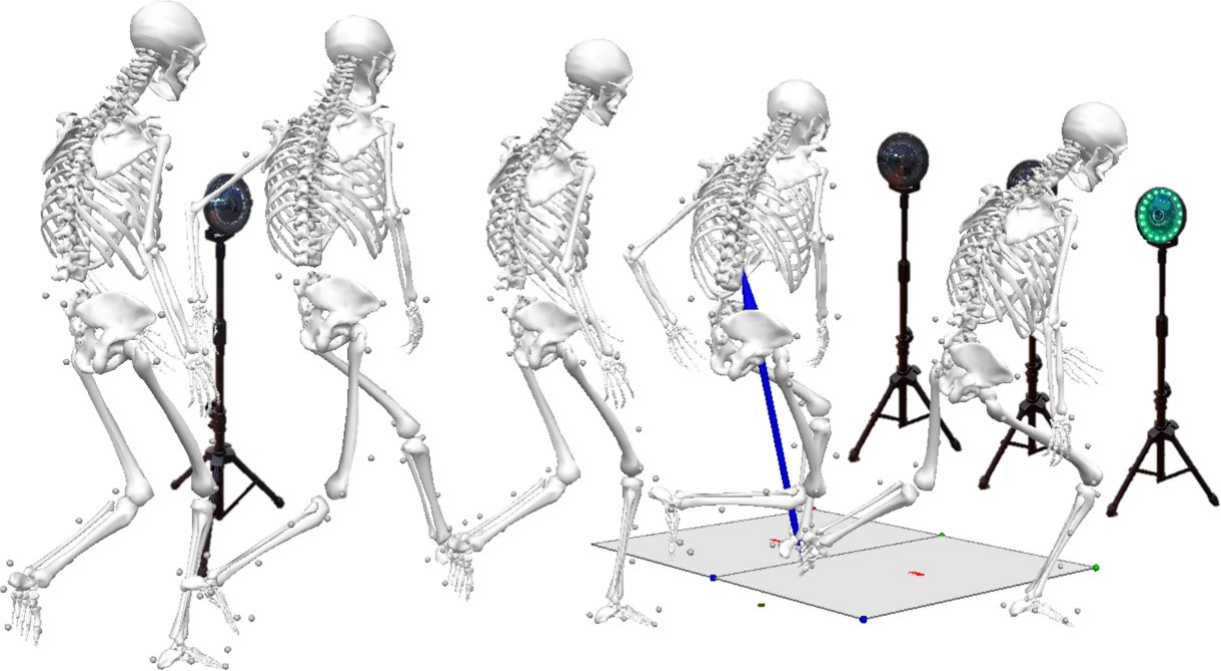
Unanticipated cutting. Example participant performing the unanticipated cutting task.

Unanticipated cutting was collected using a series of 90° sideways cuts and backpedals. Prior to each trial, participants were placed 5 meters from the force platforms and instructed to run towards them at 75% of their maximal speed. Participants were not specifically told to land on the force platform to minimize changes in their natural running style, however they were instructed which leg to land on prior to backpedaling or cutting as they approached a set of targets (FITLIGHT trainers^™,^ FITLIGHT Sports Corp., Aurora, Ontario, Canada) behind the force platforms. To create an unplanned/unanticipated task (cut vs backpedal), as they passed a trigger (2 meters in front of the force platforms) one of the directional target cues illuminated, indicating whether they were to plant and cut 90° to the side or plant and backpedal towards the starting point (Fig 2). Approach velocity of each trial was measured using timing gates (TracTronix, Lenexa, Kansas, USA) placed 2.5 meters apart (PRE: 2.8 ± 0.25m/s; PUB: 2.84 ± 0.34m/s; POST: 2.98 ± 0.34m/s). Three trials of cutting were collected on each leg (meaning they would plant on their right leg and cut 90° to the left for one set of trials, and the opposite for another set). If the participant cut or backpedaled on the incorrect leg, or if they did not fully land on the force platform, the trial was repeated, though the order of the collection of cuts and backpedals appeared randomly to each participant. Only the cutting and drop vertical jump tasks were analyzed for this study.

### Analysis

For each task, a landing phase was defined as the moment when the participant landed on the force platform (vertical ground reaction force (GRF) > 10 N) until the moment they left the platform (vertical GRF < 10 N). Marker trajectories and GRF were filtered at 12 Hz and used to calculate joint moments using inverse dynamics in Visual 3D (v6, C-Motion Inc.) [11]. Net external knee moments are described in this article with negative values representing flexion and abduction based on the analysis convention. Knee moments were analyzed unnormalized (Nm) as well as normalized to body mass (Nm/kg). Biomechanical variables of interest included peak frontal and sagittal plane knee motion and moment of the dominant limb.

Statistical analyses were performed in R programming language (version 3.6.0). A mixed-design ANOVA with the between group factor of maturation (PRE, PUB, POST) and within group factor of task (DVJ, CUT) were used in the statistical design (p<0.05). Pairwise post-hoc analyses were used if significant interactions or main effects were observed.

## Results

### Knee abduction

**Fig 3:**
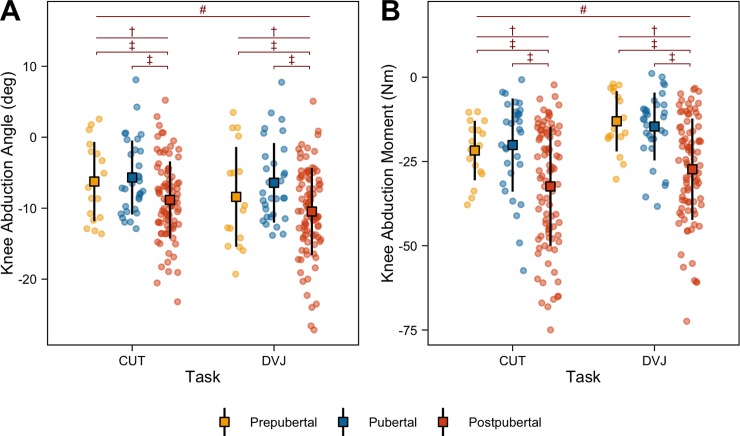
Knee abduction. Peak knee abduction angle (A) and moment (B) during the CUT and DVJ tasks for PRE, PUB, and POST groups. Boxes represent mean values with standard deviation bars. Statistically significant (p < 0.05) main effects of task (#), main effects of maturation (†), and within task differences (‡) are represented above the data.

Peak knee abduction angles during the CUT (PRE: -6.2° [95% CI: -8.8, -3.6]; PUB: -5.7° [95% CI: -7.4, -3.9]; POST: -8.8° [95% CI: -9.9, -7.7]) and DVJ (PRE: -8.4° [95% CI: -11.5, -5.2]; PUB: -6.4° [95% CI: -8.4, -4.4]; POST: -10.5° [95% CI: -11.8, -9.2]) are detailed in Fig 3A. Main effects of maturation (p = 0.005) and task (p < 0.001) were identified. Within tasks, POST exhibited significantly greater peak abduction angles compared to PUB (p < 0.001) and PRE (p = 0.003). Overall, each maturational group exhibited greater peak knee abduction angles during the double leg DVJ compared to the single leg CUT. Fig 3B details the peak knee abduction moments during the CUT (PRE: -21.7Nm [95% CI: -25.9, -17.9]; PUB: -20.1Nm [95% CI: -25.1, -15.5]; POST: -32.4Nm [95% CI: -35.9, -28.9]) and DVJ (PRE: -13.1Nm [95% CI: -17.2, -9]; PUB: -14.6Nm [95% CI: -18.1, -11.4]; POST: -27.3Nm [95% CI: -30.4, -24.2]). Main effects of maturation (p < 0.001) and task (p < 0.001) were found. Like the abduction motion, POST exhibited greater peak knee abduction moments compared to PUB (p < 0.001) and PRE (p < 0.001) within tasks. Opposite to the knee abduction motion, peak knee abduction moments during the CUT were greater compared to the DVJ across each maturational group.

### Knee flexion

**Fig 4:**
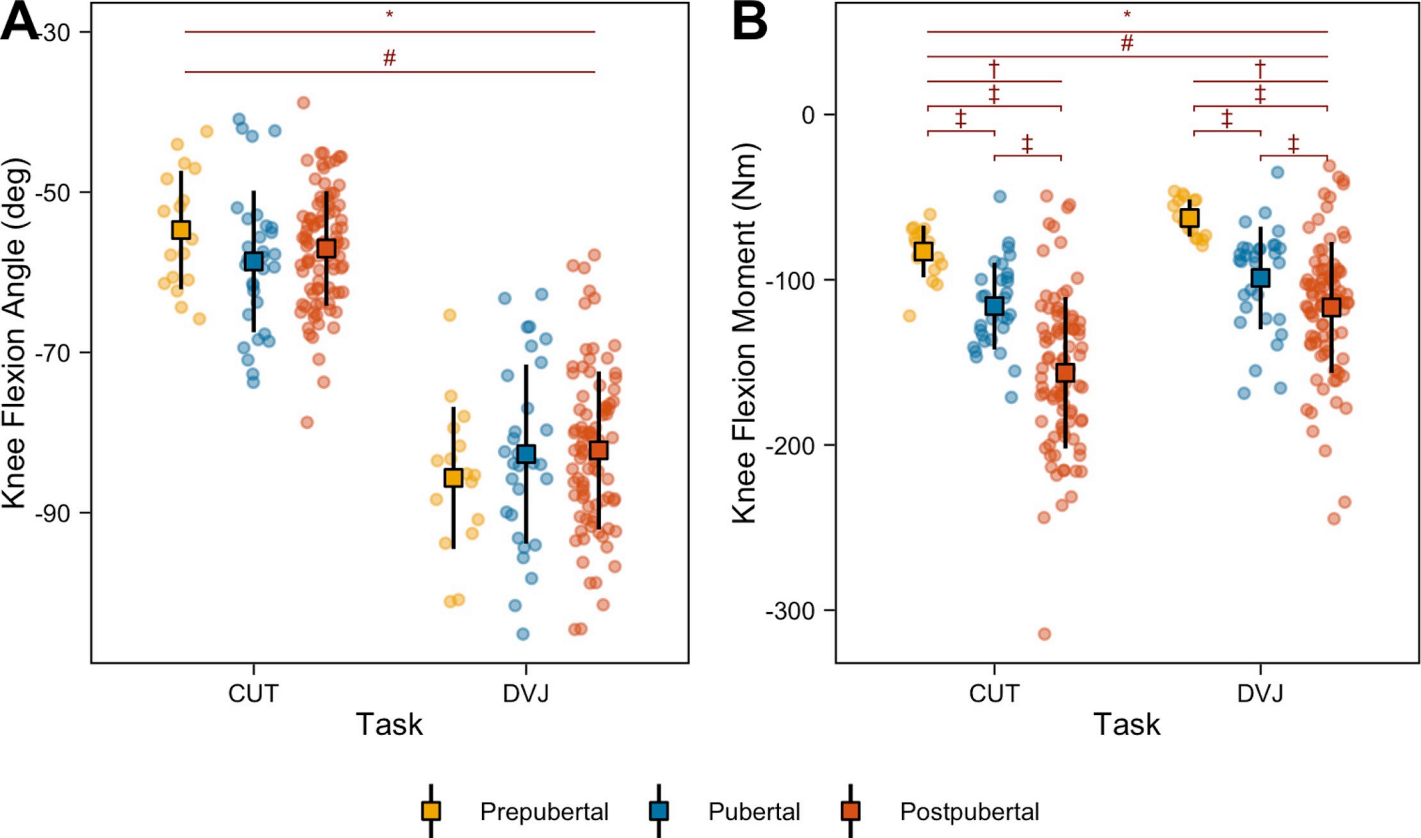
Knee flexion. Peak knee flexion angle (A) and moment (B) during the CUT and DVJ tasks for PRE, PUB, and POST groups. Boxes represent mean values with standard deviation bars. Statistically significant (p < 0.05) interactions between maturation and task (*), main effects of task (#), main effects of maturation (†), and within task significant differences (‡) are represented above the data.

Fig 4A details the peak knee flexion angles during the CUT (PRE: -54.7° [95% CI: -58, -51.4]; PUB: -58.7° [95% CI: -61.7, -55.5]; POST: -57.0° [95% CI: -58.5, -55.6]) and DVJ (PRE: -85.7° [95% CI: -89.8, -81.5]; PUB: -82.7° [95% CI: -86.5, -78.8]; POST: -82.2° [95% CI: -84.2, -80.2]). While a significant interaction was identified between maturation and task (p = 0.045), post hoc analyses did not identify a statistically significant effect of maturation in the CUT (p = 0.232) or DVJ (p = 0.436). However, as expected with landing compared to cutting, there was a main effect of task for knee flexion angle (p < 0.001), indicating that participants within each maturational group exhibited greater knee flexion during the DVJ compared to the CUT (PRE, PUB, POST: p < 0.001). Peak knee flexion moments during the CUT (PRE: -82.9Nm [95% CI: -90.5, -75.8]; PUB: -116.0Nm [95% CI: -125.0, -107.0]; POST: -156.3Nm [95% CI: -166.0, -147.0]) and DVJ (PRE: -62.6Nm [95% CI: -67.8, -57.5]; PUB: -98.9Nm [95% CI: -110.0, -88.8]; POST: -116.8Nm [95% CI: -125.0, -109.0]) are outlined in Fig 4B. A significant interaction was identified between maturation and task (p < 0.001). During the CUT, POST and PUB performed the task with greater peak knee flexion moments compared to PRE (p < 0.001), as well as POST compared to PUB (p < 0.001). Similarly, POST and PUB during the DVJ exhibited significantly greater peak knee flexion moments compared to the PRE (p < 0.001) as well as the POST compared to the PUB (p = 0.002). Between tasks, each group exhibited significantly greater peak knee flexion moments during the CUT compared to the DVJ (p < 0.001).

### Normalized knee moments

Significant main effects of maturation (p = 0.045) and task (p < 0.001) still existed for normalized peak knee abduction moment during the CUT (PRE: -0.65Nm/kg [95% CI: -0.8, -0.5]; PUB: -0.47Nm/kg [95% CI: -0.6, -0.4]; POST: -0.58Nm/kg [95% CI: -0.6, -0.5]) and DVJ (PRE: -0.39Nm/kg [95% CI: -0.5, -0.3]; PUB: -0.34Nm/kg [95% CI: -0.4, -0.3]; POST: -0.50Nm/kg [95% CI: -0.6, -0.4]). Within tasks, POST maintain greater normalized knee abduction moments compared to PUB (p = 0.002), however were not statistically different from PRE following normalization (p > 0.05). Consistent with unnormalized moment data, the CUT task elicited overall greater peak knee abduction moments compared to the DVJ. Normalized peak knee flexion moments showed similar trends as unnormalized moments for the CUT (PRE: -2.44Nm/kg [95% CI: -2.7, -2.2]; PUB: -2.67Nm/kg [95% CI: -2.8, -2.5]; POST: -2.79Nm/kg [95% CI: -2.9, -2.6]) and DVJ (PRE: -1.84Nm/kg [95% CI: -2.0, -1.7]; PUB: -2.28Nm/kg [95% CI: -2.5, -2.1]; POST: -2.1Nm/kg [95% CI: -2.2, -2.0]). A significant interaction was identified between maturation and task (p < 0.001). There was no significant main effect of maturation (p > 0.05) however a main effect of task remained (p < 0.001). Within tasks, both POST and PUB participants exhibit significantly greater normalized knee flexion moments compared to PRE (p = 0.024, p = 0.029). Within the CUT, only POST exhibited statistically greater moments compared to the PRE (p = 0.041). For the DVJ, only PUB exhibited statistically greater moments compared to the PRE (p = 0.026). Overall, each maturational group exhibited greater normalized peak knee abduction moments during the single leg CUT compared to the double leg DVJ.

## Discussion

Given the high incidence rates of ACL injury in female athletes, it is imperative to understand the role that adolescent growth and development plays on modifiable intrinsic injury risk [4, 5]. As with previous studies [10, 11], our findings confirm that knee abduction and moment during a DVJ are greater in post-pubertal females compared to those in earlier stages of maturation. These increases in knee abduction indicate an increased risk of ACL injury for this group. Hewett et al demonstrated that while females and males demonstrate greater peak knee abduction in a DVJ as they mature, only females in late adolescence stages display greater abduction (valgus) loads. This is likely due to the lack of dynamic knee control that only males regain after 91% adult stature which corresponds to peak height velocity [10].

Biomechanically, the DVJ and CUT elicited significantly different sagittal and frontal plane biomechanics. Specifically, although participants landed in a more flexed position during the DVJ, they exhibited greater knee flexion moments during the CUT likely due to the single limb support utilized in sideways cutting. This is consistent with previous research that also identified differences in the magnitude of knee flexion and abduction kinematics and moments between sidestep cutting and drop jumps [16]. Though the DVJ is often used as a common tool to indicate potential ACL injury, the biomechanics associated with the DVJ are poorly correlated with biomechanics elicited during a cutting task [16]. This indicates that both tasks should be assessed, and proper measures implemented to improve high-risk biomechanics for the optimization of ACL injury risk reduction programs.

Our results indicated similar findings in the CUT between groups where knee abduction motion and moment were greater in POST compared to the PUB and PRE female soccer players. Knee abduction moments during cutting are likely dependent on a number of factors, including initial contact technique, approach speed, cut width and the cutting angle [17]. Our participants were instructed to perform the CUT at 75% of their maximal speed. The post-pubertal players exhibited faster approach speeds than the prepubertal and pubertal players. This has the potential to elicit greater forces during play and could be an influencing factor in the greater joint moments observed during cutting.

Implementation of neuromuscular based exercise programs to reduce risk of ACL injury in young female athletes should be encouraged. By modifying high-risk lower extremity biomechanics, these types of programs have been reported to significantly reduce the risk of ACL injury [6]. This is especially true in younger females because these programs are most effective in mid teen age group categories compared to late teens and early adults [5]. Based on our results, we suggest that clinicians and other interventionists should target biomechanics during both jumping and cutting. Numerous neuromuscular based programs have been published that emphasize plyometric and technique training to address high risk biomechanics during both of these tasks [6, 14]. These programs have been shown to increase active knee stabilization and help establish safe movement patterns that translate to the field [18–20]. A potential improvement could be the inclusion of technique feedback, as it has been a recommended component of previous ACL injury prevention practices [21]. Cues from coaches and clinicians are important to correct certain mechanics, however the type of cuing may be important as motor skill learning is often best retained when cuing is given externally rather than internally [22]. ACL training programs could benefit from the use of external biofeedback, as previous studies have shown kinetic feedback during squatting to be related to landing mechanics [23]. As there is limited research on the effectiveness of such injury prevention programs to improve risk factors associated with ACL injury during cutting, it is important that future studies focus on ways to reduce potential neuromuscular deficits during both landing and cutting.

Several limitations should be considered within our study. First, a limitation in our study was that differences between maturational groups were cross-sectional in nature. However, longitudinal analyses have identified similar differences through growth and development within the DVJ. Longitudinal studies should be incorporated to examine changes in cutting throughout maturation. Second, we utilized a method to determine participant’s maturational status based on estimates of adult stature through regression equations based on longitudinal growth studies [13]. Other techniques may be utilized to classify maturational status. These may include Tanner stage classification and observational survey techniques [11, 24].

## Conclusion

Adolescent female soccer players exhibit significantly different sagittal and frontal plane biomechanics during an unanticipated single leg cutting maneuver compared to a DVJ. When comparing maturational groups, post-pubertal athletes exhibited greater peak knee flexion angle and moment as well as greater peak knee abduction angle and moment than those in earlier stages of maturation across each task. The DVJ task elicited greater peak knee flexion and abduction, however greater joint moments were observed during cutting. These biomechanical differences in knee control during two dynamic, sport-related tasks outline the need for neuromuscular training programs to reduce ACL injuries. More specifically, clinicians and coaches working with young female soccer athletes should consider the relationship of maturational status and high frontal plane knee loading. Additionally, with the high incidence of second ACL injury in this population, attention to primary risk factors should be addressed throughout the rehabilitation process in young patients following an ACL reconstruction.

## Supporting information

S1 Data (CSV)
